# Trypa-NO! contributes to the elimination of *gambiense* human African trypanosomiasis by combining tsetse control with “screen, diagnose and treat” using innovative tools and strategies

**DOI:** 10.1371/journal.pntd.0008738

**Published:** 2020-11-12

**Authors:** Joseph Mathu Ndung’u, Alain Boulangé, Albert Picado, Albert Mugenyi, Allan Mortensen, Andrew Hope, Brahim Guihini Mollo, Bruno Bucheton, Charles Wamboga, Charles Waiswa, Dramane Kaba, Enock Matovu, Fabrice Courtin, Gala Garrod, Geoffrey Gimonneau, Georgina V. Bingham, Hassane Mahamat Hassane, Inaki Tirados, Isabel Saldanha, Jacques Kabore, Jean-Baptiste Rayaisse, Jean-Mathieu Bart, Jessica Lingley, Johan Esterhuizen, Joshua Longbottom, Justin Pulford, Lingue Kouakou, Lassina Sanogo, Lucas Cunningham, Mamadou Camara, Mathurin Koffi, Michelle Stanton, Mike Lehane, Moise Saa Kagbadouno, Oumou Camara, Paul Bessell, Peka Mallaye, Philippe Solano, Richard Selby, Sophie Dunkley, Steve Torr, Sylvain Biéler, Veerle Lejon, Vincent Jamonneau, Wilfried Yoni, Zachary Katz

**Affiliations:** 1 Foundation for Innovative New Diagnostics (FIND), Geneva, Switzerland; 2 Centre International de Recherche-Développement sur l’Elevage en zone Subhumide (CIRDES), Bobo Dioulasso, Burkina Faso; 3 Centre International de Recherche Agronomique pour le Développement (CIRAD), UMR INTERTRYP, Montpellier, France; 4 Coordinating Office for Control of Trypanosomiasis in Uganda (COCTU), Entebbe, Uganda; 5 Vestergaard SA, Lausanne, Switzerland; 6 Liverpool School of Tropical Medicine (LSTM), Liverpool, United Kingdom; 7 Institut de Recherche en Elevage pour le Développement (IRED), N’Djamena, Chad; 8 Institut de Recherche pour le Développement (IRD), INTERTRYP, CIRAD, Université Montpellier, Montpellier, France; 9 Programme National de Lutte contre la Trypanosomiase Humaine Africaine, Conakry, Guinea; 10 Ministry of Health (MOH), Kampala, Uganda; 11 Institut Pierre Richet/Institut National de Santé Publique (IPR/INSP), Bouaké, Côte d’Ivoire; 12 College of Veterinary Medicine, Animal Resources and Biosecurity (COVAB), Makerere University, Kampala, Uganda; 13 Programme National d'Elimination de la THA (PNETHA), Abidjan, Côte d’Ivoire; 14 Université Jean Lorougnon Guédé (UJLoG), Daloa, Côte d’Ivoire; 15 Epi Interventions, Edinburgh, United Kingdom; 16 Programme National de Lutte contre la Trypanosomiase Humaine Africaine (PNLTHA), Chad; Universiteit Antwerpen, BELGIUM

## Introduction

*Gambiense* human African trypanosomiasis (g-HAT) is the chronic form of sleeping sickness caused by *Trypanosoma brucei gambiense* in West and Central Africa, while *Trypanosoma brucei rhodesiense* causes an acute form in eastern Africa. g-HAT is targeted for elimination as a public health problem by 2020 and 0 transmission by 2030 [1,2]. Control of g-HAT is largely based on identification and treatment of infected individuals, supplemented by control of the tsetse fly vectors [3]. There has been growing evidence that when both tsetse control and case identification activities are carried out simultaneously in the same geographies, elimination of the disease is accelerated [4–6]. Here, we describe how the Trypa-NO! Partnership is using novel and classical tools to drive g-HAT elimination in an integrated approach, progress made, lessons learnt, and future directions.

## The Trypa-NO! Partnership

### Goal

The Trypa-NO! Partnership was established in September 2016 to support National Sleeping Sickness Control Programmes (NSSCP) in Chad, Côte d’Ivoire, Republic of Guinea, and Uganda in driving elimination of g-HAT by integrating tsetse control with screening, diagnosis, and treatment of cases. The Partnership goals are to drive to 0 the annual number of g-HAT cases reported in Côte d’Ivoire and Uganda by 2020 and reduce cases by 90% in the Republic of Guinea and Chad by 2022.

### Composition and governance

The Partnership, as illustrated in S1 Fig, includes government departments and in-country partners involved in research and control of tsetse and g-HAT in respective countries, a number of international organisations, and the Bill and Melinda Gates Foundation (BMGF). Country partnership committees, which include the NSSCP oversee implementation of activities, while overall coordination of the Partnership is by a Steering Committee (SC). The SC meets quarterly to review progress and give strategic direction. An Advisory Committee, comprising of WHO and experts in disease elimination and tsetse and trypanosomiasis control, reviews reports from the Partnership and makes recommendations on the way forward.

### Implementation strategy

The Trypa-NO! Partnership strategy integrates tsetse control with screening, diagnosis, and treatment of g-HAT cases and a system of collection and transfer of data into a Central Information Repository (CIR). The data are analysed and used in developing and updating microplans that guide activities. Activities are organised in work packages (WP), each with a WP leader responsible to the SC for ensuring that microplans are followed, and important outputs shared among the Partnership and other stakeholders. The WPs include HAT screening, diagnosis, and treatment (WP1), vector control and One Health (WP2), and data, mapping, and integrated surveillance (WP3), as illustrated in S1 Fig.

### Screening, diagnosis and treatment

Diagnosis of g-HAT consists of multiple steps, including combining clinical signs with screening blood for anti-trypanosomal antibodies. Endemic villages are targeted for active medical surveys, which use the classical card agglutination test for trypanosomiasis (CATT) [7] to screen the entire population. Suspected cases in health facilities are tested individually using recently developed rapid diagnostic tests (RDT) [8,9]. Individuals found positive with any of the screening tests undergo confirmatory testing using various microscopy methods [10–12]. For patients who test positive by microscopy, a lumbar puncture is performed to determine the disease stage, and treatment is given according to WHO recommendations (pentamidine for stage 1 and NECT for stage 2 patients). In Guinea, a subset of patients were treated with either fexinidazole or acoziborole, as part of ongoing clinical trials.

Besides active and passive surveillance, and in order to ensure effective coverage of the population at risk, the Trypa-NO! Partnership has also adopted a number of alternative strategies to improve case detection in an elimination context. In these strategies, which may be referred to as “targeted reactive screening,” inhabitants neighbouring households where cases have recently been detected are screened. This is done in a number of ways, including “door-to-door” screening by technicians who walk from 1 house to another [13], or by light mobile teams that move on motorbikes, or by medical teams that concentrate on specific sites. The medical teams target the most at risk people, e.g., in “boat landing points” in Guinea, coffee/cocoa plantations in Côte d’Ivoire, or market places in Chad. A spatial follow-up of HAT cases and mapping of areas where they are likely to have been infected is used to orient medical and vector activities [14]. In addition, a geographical method called “Identification of Villages at Risk” (IVR) is implemented in historical foci and areas at risk, where the situation of g-HAT is not well known, in order to update the epidemiological situation [15]. In Côte d’Ivoire, these efforts are supplemented by medical teams that follow up and retest people who remain positive with screening tests but negative by microscopy, who would thus not be treated until parasites are demonstrated [14].

### Tsetse control

**Fig 1:**
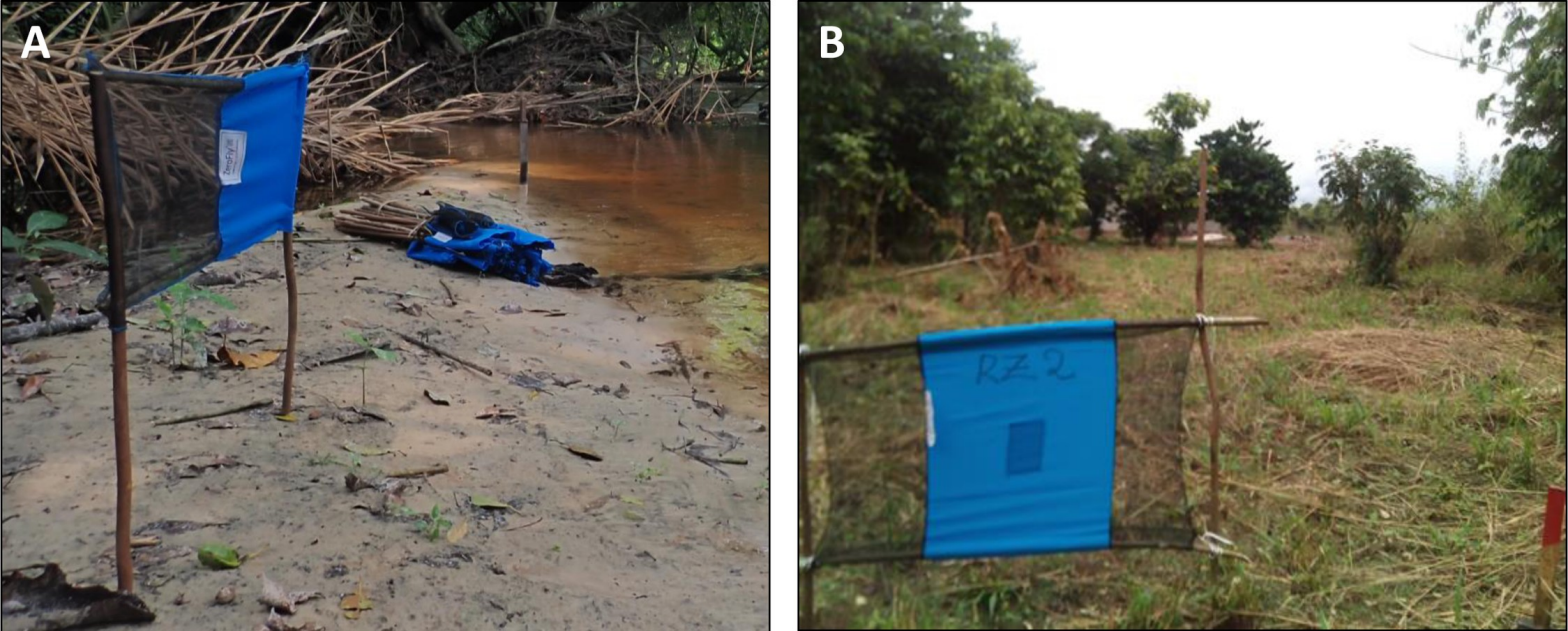
**Tiny Targets used to control (A) *G*. *fuscipes* ssp. in Central Africa and (B) *G*. *palpalis* ssp. in West Africa.** *Image credit*: (A) Sophie Dunkley, LSTM; (B) Fabrice Courtin, IRD.

A variety of insecticide-based methods have been used to control tsetse [16]. Previously, methods used to attract and kill the vectors of *rhodesiense* HAT (r-HAT) and animal African trypanosomiasis (AAT) proved prohibitively expensive to control g-HAT vectors [17] due to differences in their host-seeking behaviour, but work in Côte d’Ivoire in the 1990s showed that this approach can be successful and cost effective [18]. Subsequent research led to development of “Tiny Targets”, small (0.5 m × 0.25 m for *Glossina fuscipes* ssp.) [19] or medium-sized (0.75 m × 0.50 m for *Glossina palpalis* ssp.) [20] insecticide-impregnated panels of cloth that are cheap to produce and deploy, making the incremental costs of adding tsetse control to screening humans affordable and feasible at scale and across a wide range of epidemiological settings (Fig 1). Initial trials in Uganda [21], Guinea [5], and Chad [6] showed that Tiny Targets rapidly reduced tsetse densities by 60% to 80% and the incidence of g-HAT, with at least 70% of the reduction in g-HAT incidence attributed to their use [5,6]. Following the trials, tsetse control has been implemented at scale in the active g-HAT foci of Guinea from 2012, Chad from 2013, Uganda from 2014, and Côte d’Ivoire from 2017.

The Trypa-NO! Partnership has adopted 5 features of tsetse control in all countries. First, tsetse control is only carried out in areas where g-HAT cases have been reported recently, and hence clear evidence of ongoing local transmission of *T*.*b*. *gambiense* (active foci). Second, initial geographical surveys are carried out and the tsetse control area mapped. Third, local communities are sensitised on the use of targets for tsetse control. Fourth, locally recruited people deploy the targets, and the location of every target is georeferenced. These teams are trained by specialists from the Trypa-NO! Partnership and the national programmes. Using local people to deploy targets has been proved cost effective and sustainable [17–19]. Fifth, tsetse suppression is monitored through regular entomological surveys. Integrating data on the distribution and abundance of g-HAT cases, targets, and tsetse allows planners to adjust the programme according to results.

## Use of data to guide implementation

### Central information repository

The CIR is a database that consolidates in 1 location, data, and materials that are of relevance for analysing and understanding programme activities, evaluating progress towards the Partnership’s goals, and guiding implementation of activities, as illustrated in S2 Fig. Data on vector control and medical activities included in the CIR are cleaned and enriched by addition of geo-coordinates if the data are not already georeferenced, and through data sharing agreements (DSAs), made available to anyone that may be interested in using it, such as modellers. The CIR is hosted by the Foundation for Innovative New Diagnostics (FIND) on behalf of the partnership under a DSA with all members of the Partnership. The DSA specifies that all data in the CIR that originated from a partner country remain property of that country and that country partners hold the rights to determine how their data are used and by whom and have a right to veto any particular use of the data. Thus, alongside the CIR, Trypa-NO! maintains a dialogue between the country that originated data and the users of the data. In the event that the Partnership comes to an end, all resources in the CIR will be transferred to the respective countries.

### Mapping and microplanning

The Trypa-NO! Partnership uses the results and analysed data from the CIR to guide programmatic decision-making and to revise and update the planned activities, which are summarised into microplans. The microplans comprise a map showing the region where activities are ongoing and a Gantt chart describing planned activities (S1–S4 Appendices).

### Modelling

Linkages have been established with HAT modelling groups (1) to ensure that they have access to the data they need, in a format that they require; and (2) to address questions that are pertinent to the activities of Trypa-NO! that could be addressed by mathematical, statistical, or economic modellers.

## Achievements

### Screening, diagnosis and treatment

Between 2017 and 2019, 350 g-HAT cases were identified after screening 442,027 people (Table 1). It should be noted that these are the combined results from all activities in the project areas, including others that were funded from other sources. The majority of cases were in Guinea, followed by Chad. In Côte d’Ivoire, 2 cases were detected during targeted reactive screening (follow-up of seropositive suspects) and 3 during passive surveillance. In Uganda, a civil conflict in South Sudan that started in 2016 led to a massive influx of refugees from HAT endemic regions. Majority of the refugees stay in well-defined settlements in various parts of the country, while others have integrated with the local communities, which posed a risk of introducing more HAT cases in Uganda. Trypa-NO! responded by increasing the number of health facilities screening for the disease and the frequency of active screening, with more than 95,000 people screened in 2018 alone. During the period reported on here, 4 HAT cases were identified among refugees in this area.

**Table 1:** Number of people screened actively and passively for g-HAT and cases identified in the 4 countries in the Trypa-NO! Partnership from 2017 to 2019.

Country	Screening strategy							
	Active		Reactive		Passive		Total	
	Number tested	HAT cases	Number tested	HAT cases	Number tested	HAT cases	Number tested	HAT cases
Chad	129,520	41	0	0	13,763	16	143,283	57
Côte d'Ivoire	32,711	0	8,280	2	4,045	3	45,036	5
Guinea	31,720	103	17,442	72	23,705	107	72,867	282
Uganda	137,146	1	24,430	0	19,265	5	180,841	6
**Total**	331,097	145	50,152	74	60,778	131	42,027	350

g-HAT, *Gambiense* human African trypanosomiasis.

### Tsetse control

**Fig 2:**
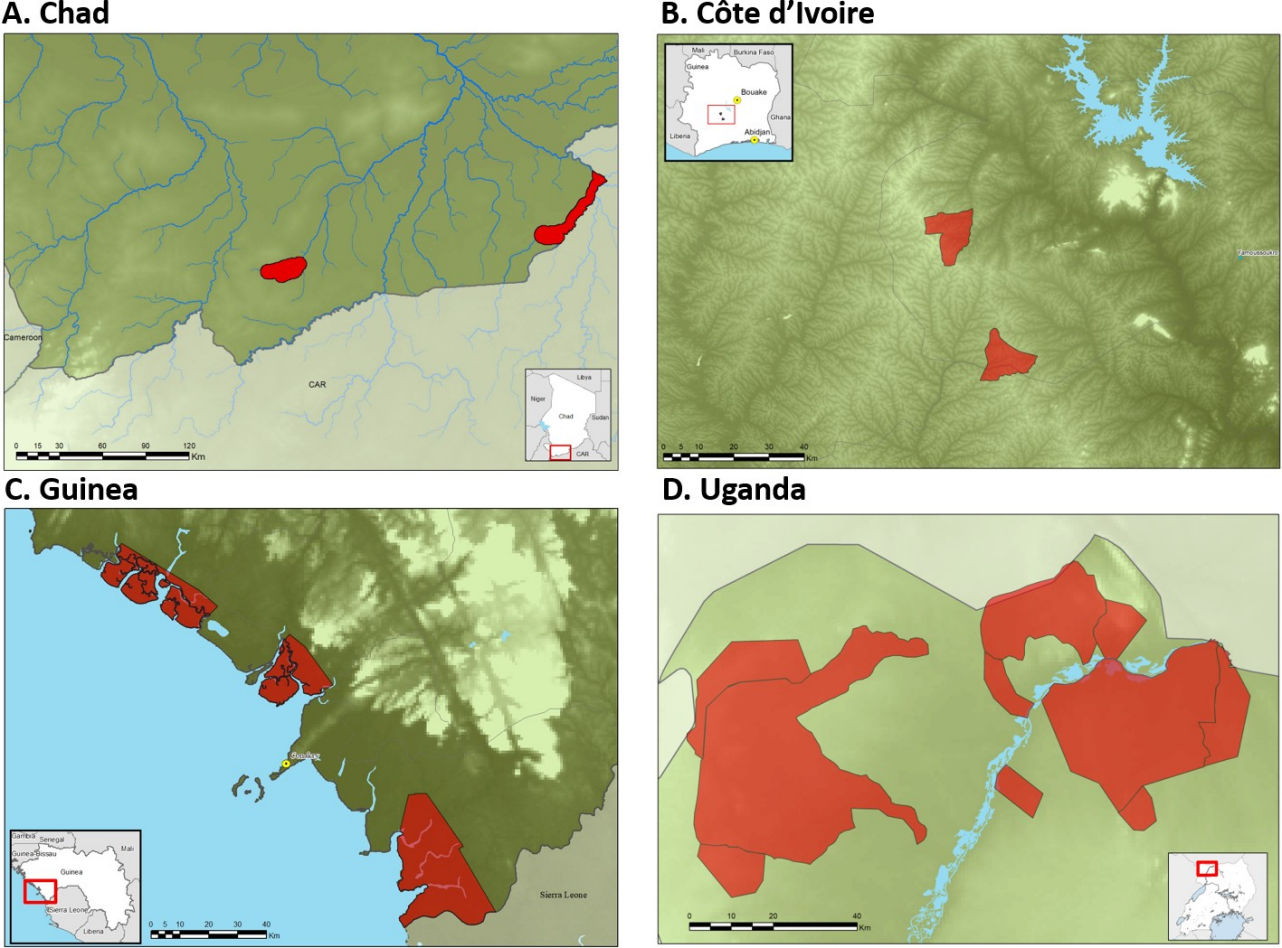
Regions where Tiny Targets (red areas) are deployed in countries in the Trypa-NO!. Partnership Fig 2A areas of Tiny Target deployments in Chad; Fig 2B Côte d’Ivoire; Fig 2C Guinea; and Fig 2D Uganda.

In the 4 countries in the Trypa-NO! Partnership, Tiny Targets are deployed across an estimated aggregate area of nearly 7,000 km^2^, protecting over a million people (Table 2 and Fig 2). This includes a range of agroecological settings, from wetlands in Mandoul and gallery forest in Maro, all along river Grande Sido in southern Chad, mangrove swamps in Guinea, to densely populated farming areas in Uganda and Côte d’Ivoire. The largest operation is in Uganda, where Tiny Targets were first deployed over 250 km^2^ in 2011 [21]. Currently, Uganda deploys approximately 42,000 targets/year across nearly 4,000 km^2^, achieving >80% reduction in tsetse densities, and protecting more than a million people. At its maximum during the Trypa-NO! project, the Uganda programme was covering an area of approximately 4,900 km^2^, which included vector control as an emergency response to the South Sudan refugee crisis.

**Table 2:** Population protected and areas covered (km^2^) with Tiny Targets in countries in the Trypa-NO! Partnership. Areas covered are estimated from Fig 2 and populations estimated from WorldPop (https://www.worldpop.org).

Country	Foci	Population protected	Area covered (km^2^)
Chad	Mandoul and Maro	80,000	960
Côte d’Ivoire	Bonon and Sinfra	170,000	250
Guinea	Boffa, Dubreka, and Forecariah	200,000	1,900
Uganda	West Nile	1,139,000	3,900
**Total**		**1,519,000**	**7,600**

In Chad, up to 5,000 Tiny Targets have been deployed annually since 2014 in the 2 most active foci in the country, contributing to a progressive decrease in incidence of g-HAT, from more than 200 cases/year before vector control started to less than 10/year today [2]. Similarly, in Côte d’Ivoire, deployment of up to 3,000 Tiny Targets annually in the Bonon and Sinfra foci starting from 2016 has reduced tsetse densities by more than 95%, protecting more than 170,000 inhabitants over an area of 250 km^2^. In Guinea, vector control, initially implemented from 2012 in part of the Boffa focus, was extended to all 3 active foci, with up to 20,000 Tiny Targets deployed each year. This has contributed to a sharp decrease in disease incidence and played a critical role in combating an upsurge of g-HAT cases that occurred (2016 to 2017) after the Ebola crisis [22].

### Conclusions

The results obtained after just over 3 years of integrated medical and vector control activities in Côte d’Ivoire, Chad, Guinea, and Uganda indicate that the strategy implemented by the Trypa-NO! Partnership is effective, as the original objectives have largely been met. Indeed, the number of g-HAT cases reported annually in Côte d’Ivoire and Uganda is close to 0, with an impressive reduction in the number reported in Guinea (50% reduction from 2017) and in Chad (68.5% reduction from 2016), increasing the prospects of reaching the goal of 90% reduction by 2022. Although the contribution of other factors such as the Ebola crisis in Guinea, changes in land use, and control activities supported through other projects should not be ignored, the Trypa-NO! Partnership is making significant contributions to the NSSCP and WHO goals of eliminating g-HAT in these countries, as evidenced by both a progressive reduction in g-HAT cases and a sharp and sustained decline in fly densities in project areas.

A key contributor to the success of Trypa-NO! is the integration of medical and vector control activities, using data collected during interventions to guide and inform planning of subsequent activities. Whenever g-HAT cases are identified in areas that have not reported cases recently, a rapid response is implemented to prevent further transmission, including reactive screening and intensified vector control. For example, the strong and almost immediate responses to the few g-HAT cases identified in Côte d’Ivoire and among refugees in Uganda may have prevented the occurrence of many other cases in refugee settlements and in neighbouring communities. The strategies described here, of combining medical surveillance with vector control using novel and classical tools for g-HAT elimination, have great potential for replication and scaling up in other foci with comparable epidemiological status, and may provide information to guide policies towards driving and sustaining elimination of the disease.

### Future direction

As the Trypa-NO! Partnership makes progress towards g-HAT elimination, it continuously adapts interventions in response to a rapidly evolving epidemiological context and adjusts activities by focusing efforts to where there is a need. For example, in Uganda, the number of health facilities screening for g-HAT was adjusted based on epidemiological and demographic data, from 152 to 174 at the height of the refugee crisis in 2017, then to 51 at the end of 2018. Likewise, in Côte d’Ivoire, the strategy is shifting from mainly active screening to targeted reactive screening and an increase in facilities conducting passive screening. These adjustments are likely to ensure that the strategies are sustainable in a post-elimination scenario, while keeping the population at risk under surveillance. Following the principle that vector control is implemented only where there is evidence of recent transmission, we are testing a strategy of discontinuing the deployment of Tiny Targets when there has been a period of 5 years without any cases of g-HAT. This strategy is currently being tested in Maracha district of Uganda where targets were first deployed in 2011 and the “last” case of g-HAT was reported in 2012. Pursuing community engagement in vector control and sustaining the progressive integration of new diagnosis (RDTs) and treatment (oral drugs) tools in the healthcare system will be key to achieving sustainable elimination of g-HAT in these countries and progress towards the 2030 “zero transmission” goal.

Some challenges remain and are addressed as they arise. We are conscious of the need to study potential cryptic reservoirs (humans and animals) that could threaten elimination [23]. Preliminary data collected in the framework of the Trypa-NO! Partnership confirm that a better understanding of the contribution of such potential reservoirs in the epidemiology of g-HAT through a “One Health” approach is mandatory to advance towards the interruption of transmission. Due to the challenges brought by the Ebola outbreak in Guinea, the Partnership also intends to sustain intensified screening and vector control, at least up to 2022, and extend activities based on availability of funding. In some places, it is likely that vector control will be scaled back but monitoring sustained, and a capacity to rapidly reintroduce vector control will be established and maintained. Strategies may also have to be adapted in response to the challenges presented by the ongoing Coronavirus Disease 2019 (COVID-19) pandemic. Indeed, active screening has already been scaled back as it requires gathering people in large numbers, which has been discouraged during the pandemic by WHO [24]. Fortunately, a planned intensified use of the 2 new technologies deployed in this Partnership, including RDTs and Tiny Targets, for passive screening and vector control could prevent a surge in cases during the crisis.

## Supporting information

S1 Fig An illustration of the operational structure of the Trypa-NO! Partnership. (PPTX)

S2 Fig A chart illustrating the flow and management of data and its use in the Trypa-NO! Partnership project. (TIF)

S1 Appendix Example of microplans for Chad, comprising the whole country, with separate specific plans for the Mandoul and Maro HAT foci. The microplans comprise of a map showing the region where activities are ongoing and a Gantt chart describing planned activities.
(PPTX)

S2 Appendix Example of microplans for Côte d’Ivoire, comprising the whole country, with a separate specific plan for the Bonon and Sinfra foci. The microplans comprise of a map showing the region where activities are ongoing and a Gantt chart describing planned activities.
(PPTX)

S3 Appendix Example of a microplan for Guinea. The microplan comprises of a map showing the region where activities are ongoing and a Gantt chart describing planned activities.
(JPG)

S4 Appendix Example of a microplan for Uganda. The microplan comprises of a map showing the region where activities are ongoing and a Gantt chart describing planned activities.
(JPG)
