# Supplementary material for: Trypa-NO! contributes to the elimination of gambiense human African trypanosomiasis by combining tsetse control with “screen, diagnose and treat” using innovative tools and strategies
Source: PLoS Negl Trop Dis. 2020 Nov 12;14(11):e0008738. doi: 10.1371/journal.pntd.0008738 (PMC7660505; doi:10.1371/journal.pntd.0008738)
Supplement: S1 Fig — (PPTX) [file pntd.0008738.s001.pptx]

## Slide 1
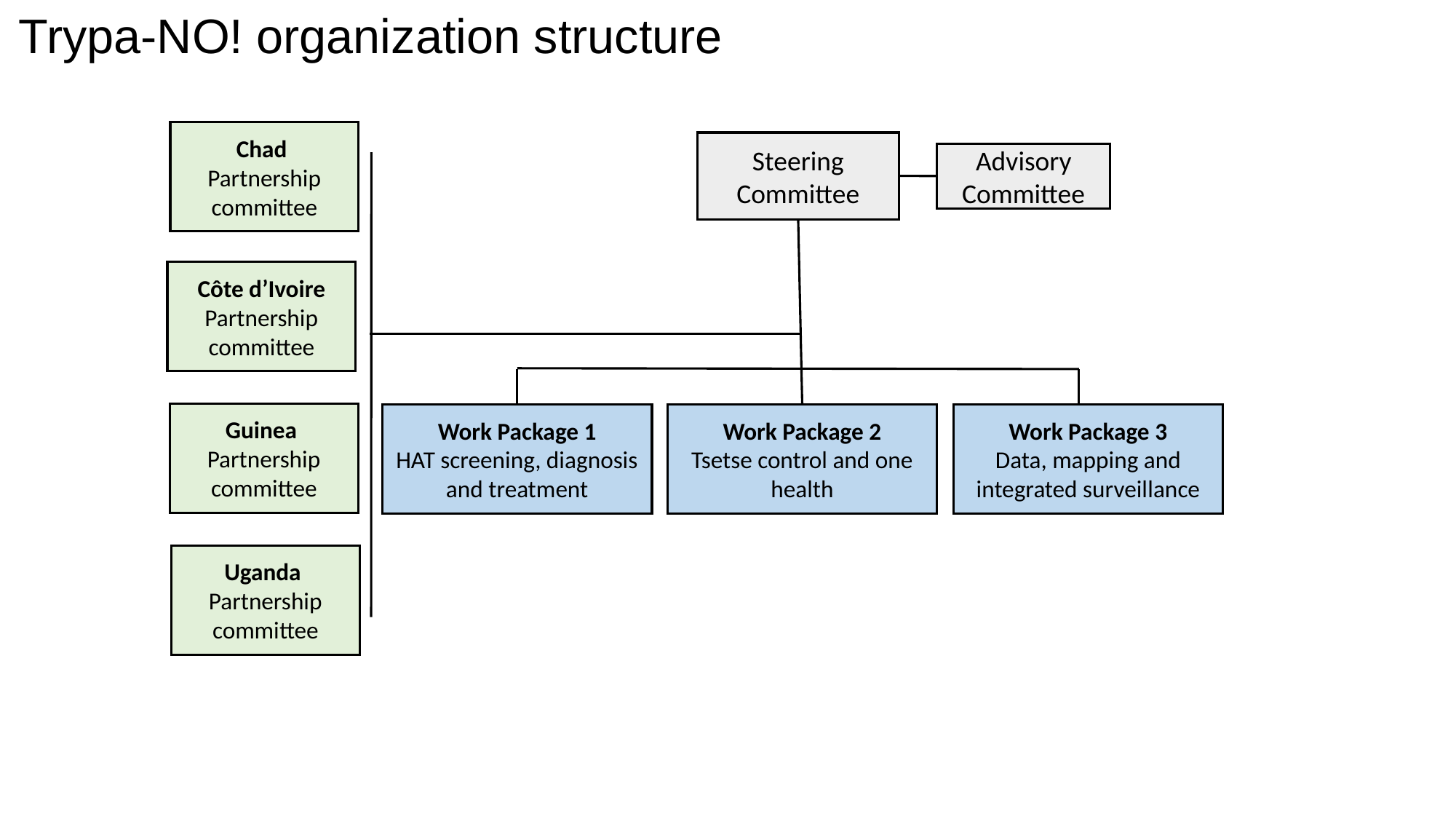

Trypa-NO! organization structure
Chad
Partnership committee
Steering Committee
Advisory Committee
Côte d’Ivoire
Partnership committee
Guinea
Partnership committee
Work Package 1
HAT screening, diagnosis and treatment
Work Package 2
Tsetse control and one health
Work Package 3
Data, mapping and integrated surveillance
Uganda
Partnership committee
