# Supplementary figures and images for: Trypa-NO! contributes to the elimination of gambiense human African trypanosomiasis by combining tsetse control with “screen, diagnose and treat” using innovative tools and strategies
Source: PLoS Negl Trop Dis. 2020 Nov 12;14(11):e0008738. doi: 10.1371/journal.pntd.0008738 (PMC7660505; doi:10.1371/journal.pntd.0008738)

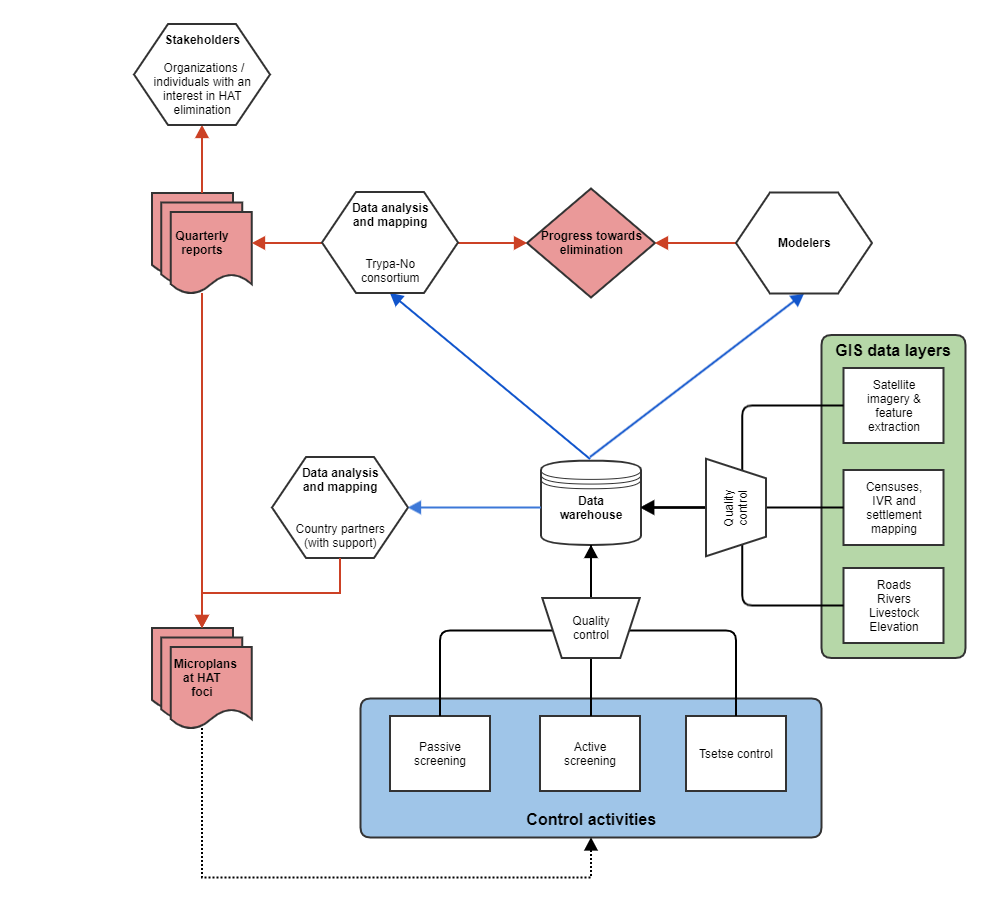

Supplement: S2 Fig — (TIF) [file pntd.0008738.s002.tif]

## Slide 1
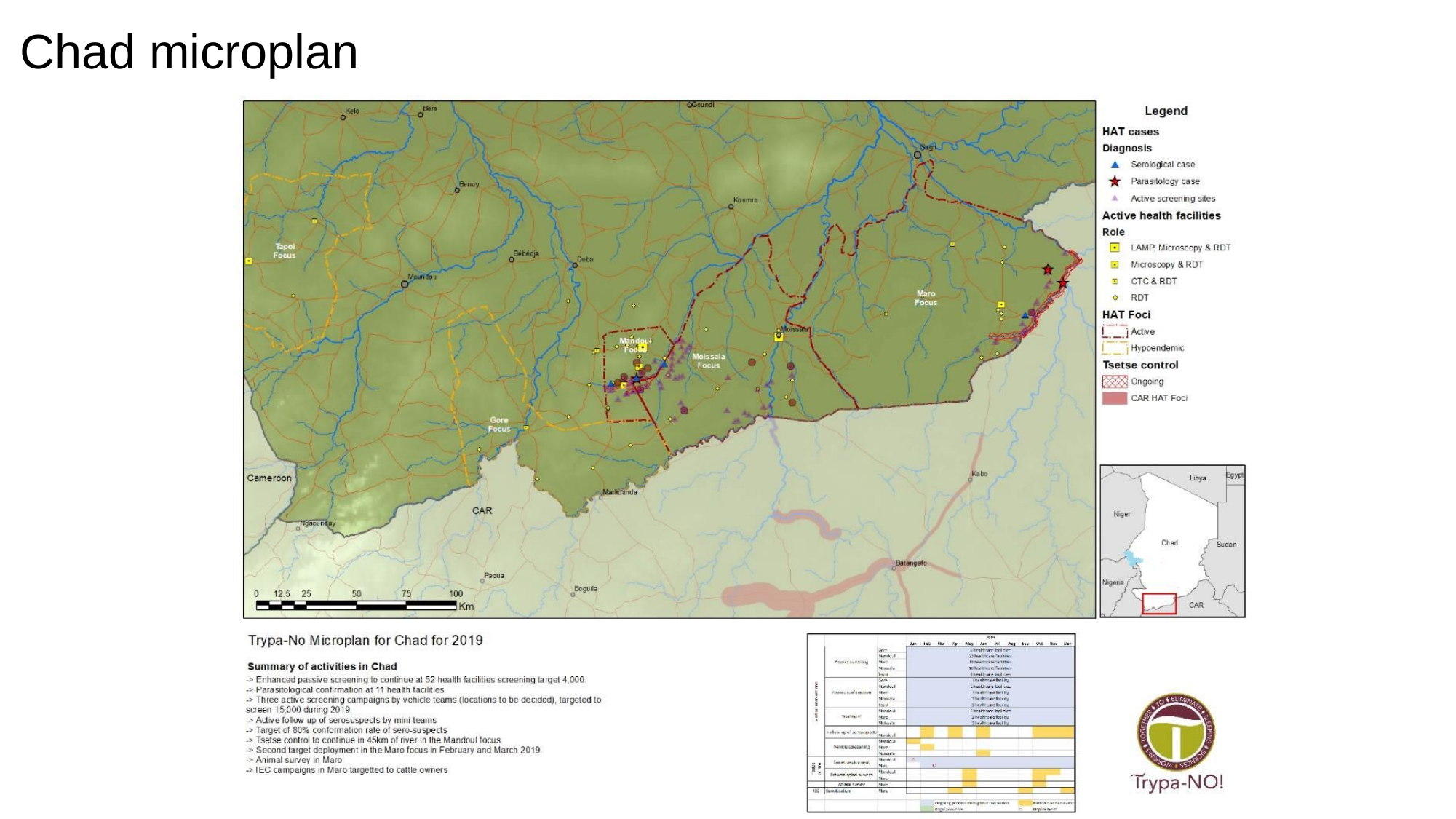

# Chad microplan

## Slide 2
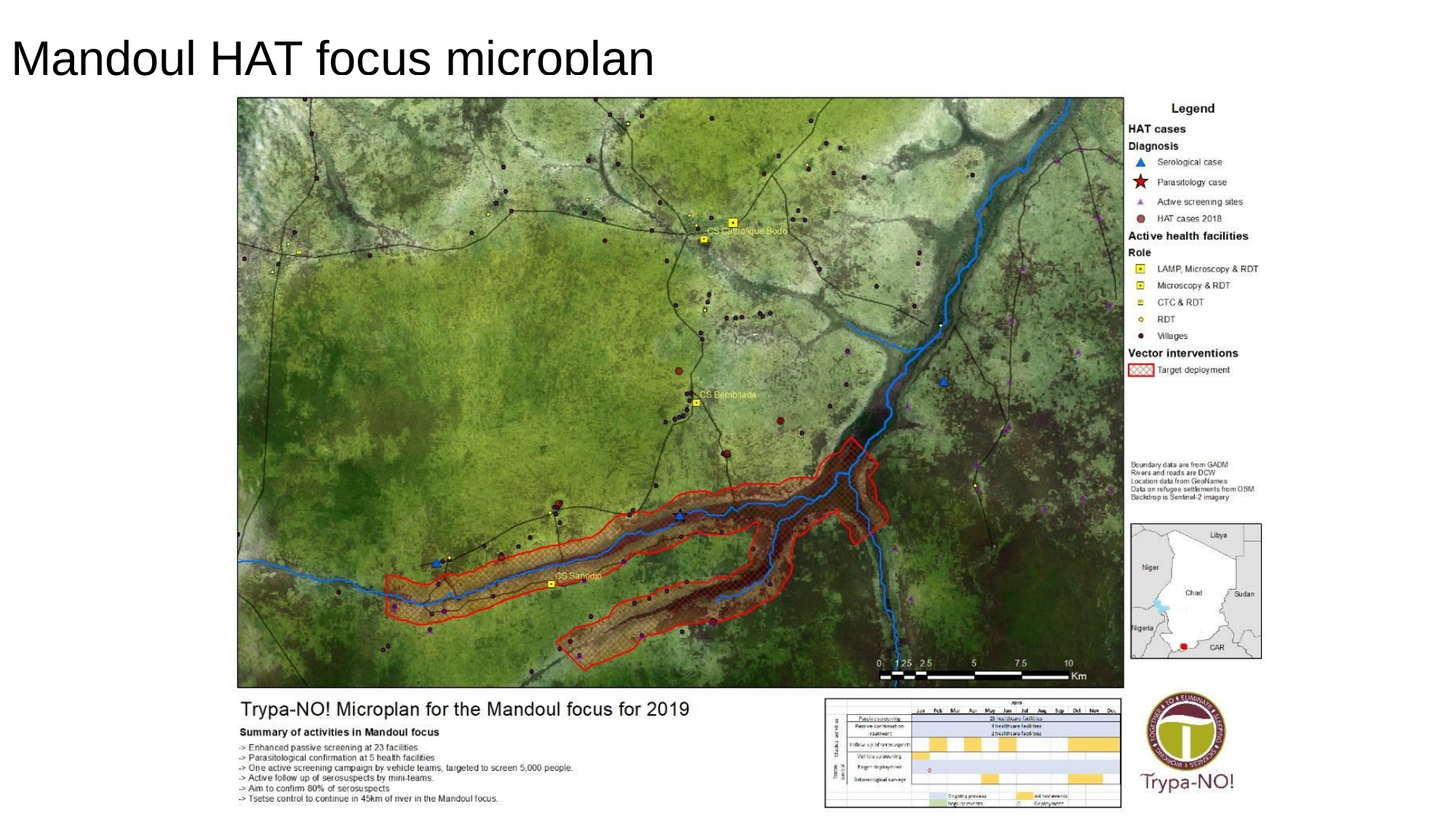

# Mandoul HAT focus microplan

## Slide 3
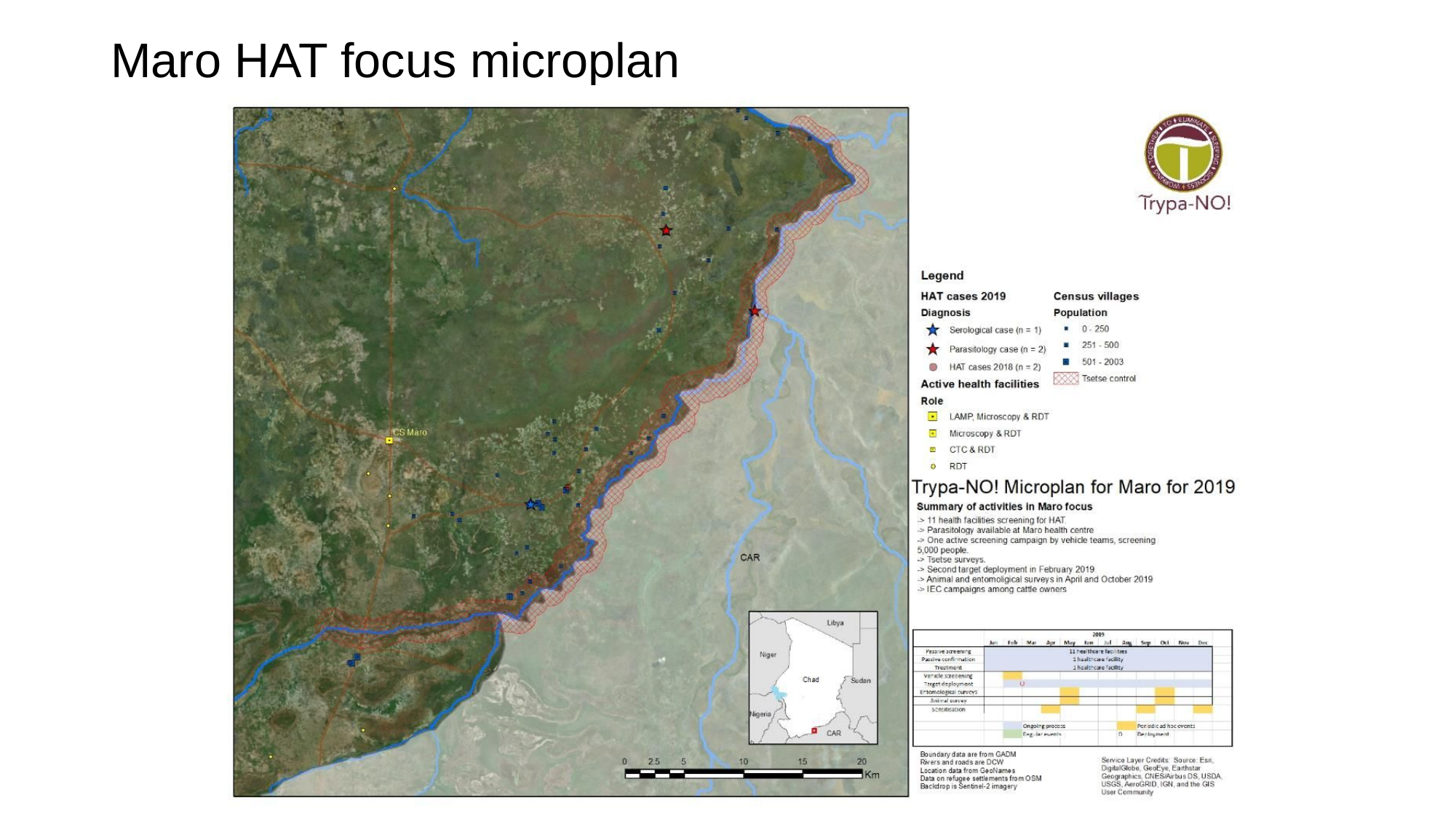

# Maro HAT focus microplan

Supplement: S1 Appendix — The microplans comprise of a map showing the region where activities are ongoing and a Gantt chart describing planned activities. (PPTX) [file pntd.0008738.s003.pptx]

## Slide 1
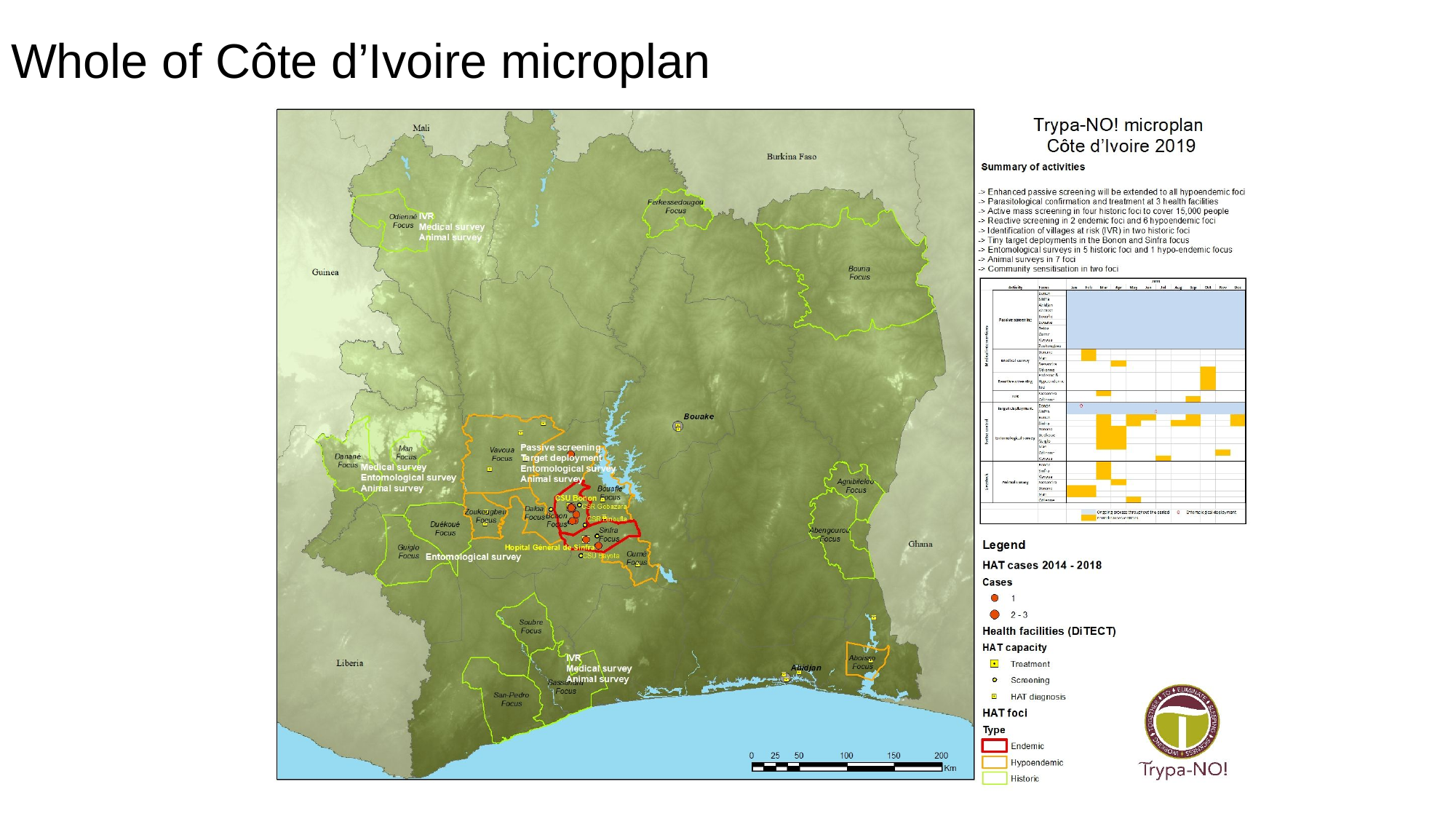

# Whole of Côte d’Ivoire microplan

## Slide 2
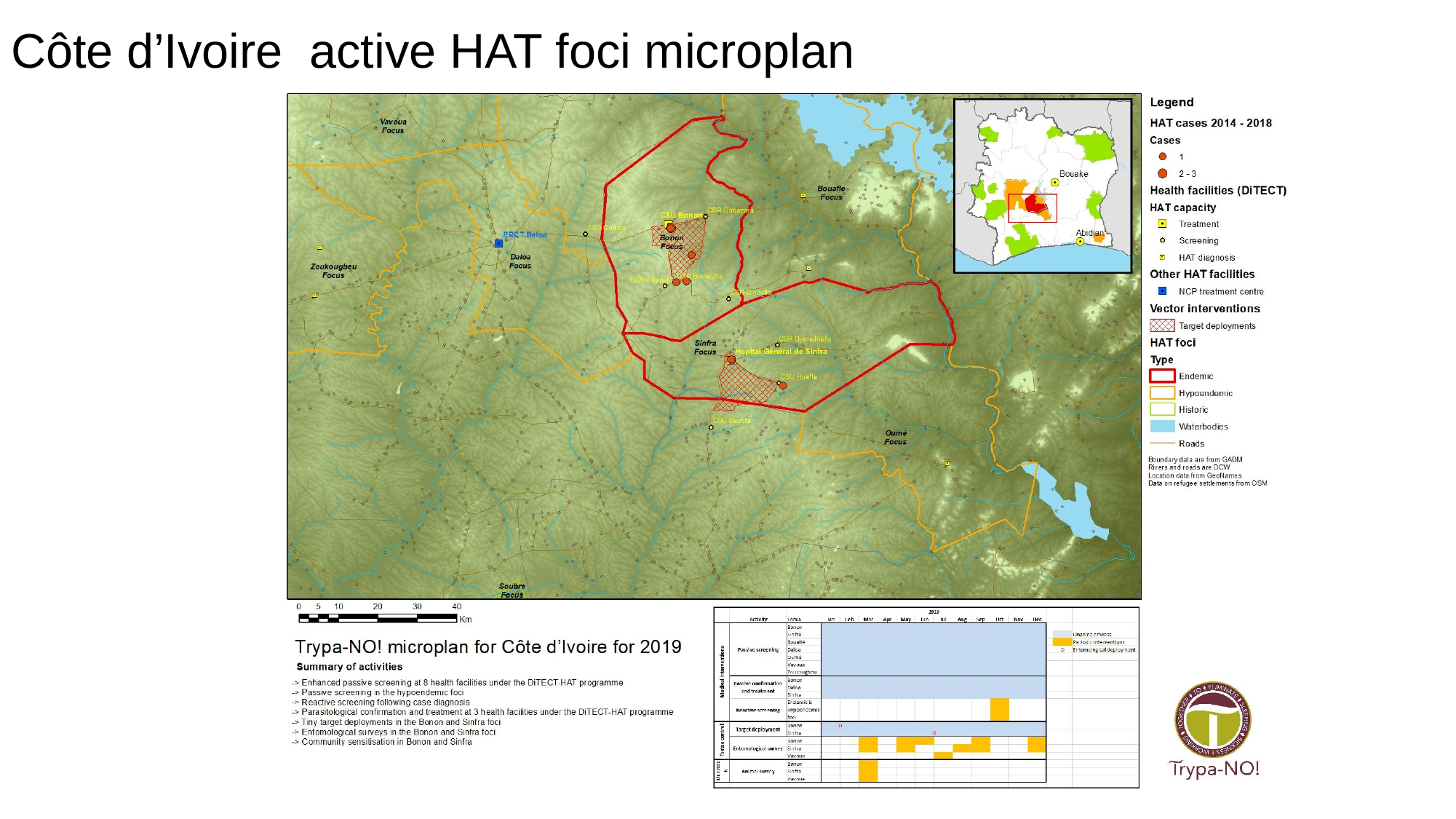

# Côte d’Ivoire active HAT foci microplan

Supplement: S2 Appendix — The microplans comprise of a map showing the region where activities are ongoing and a Gantt chart describing planned activities. (PPTX) [file pntd.0008738.s004.pptx]

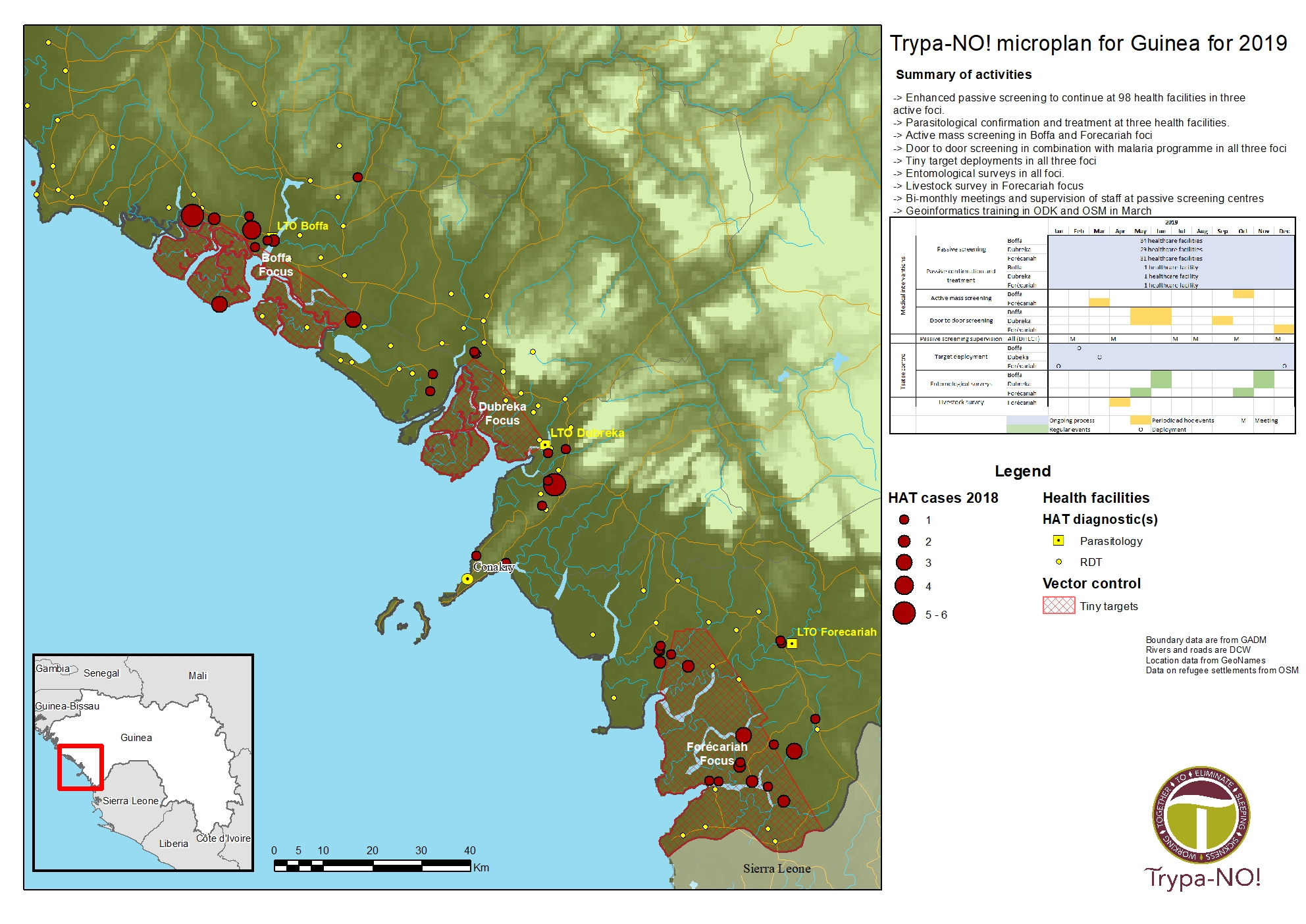

Supplement: S3 Appendix — The microplan comprises of a map showing the region where activities are ongoing and a Gantt chart describing planned activities. (JPG) [file pntd.0008738.s005.jpg]

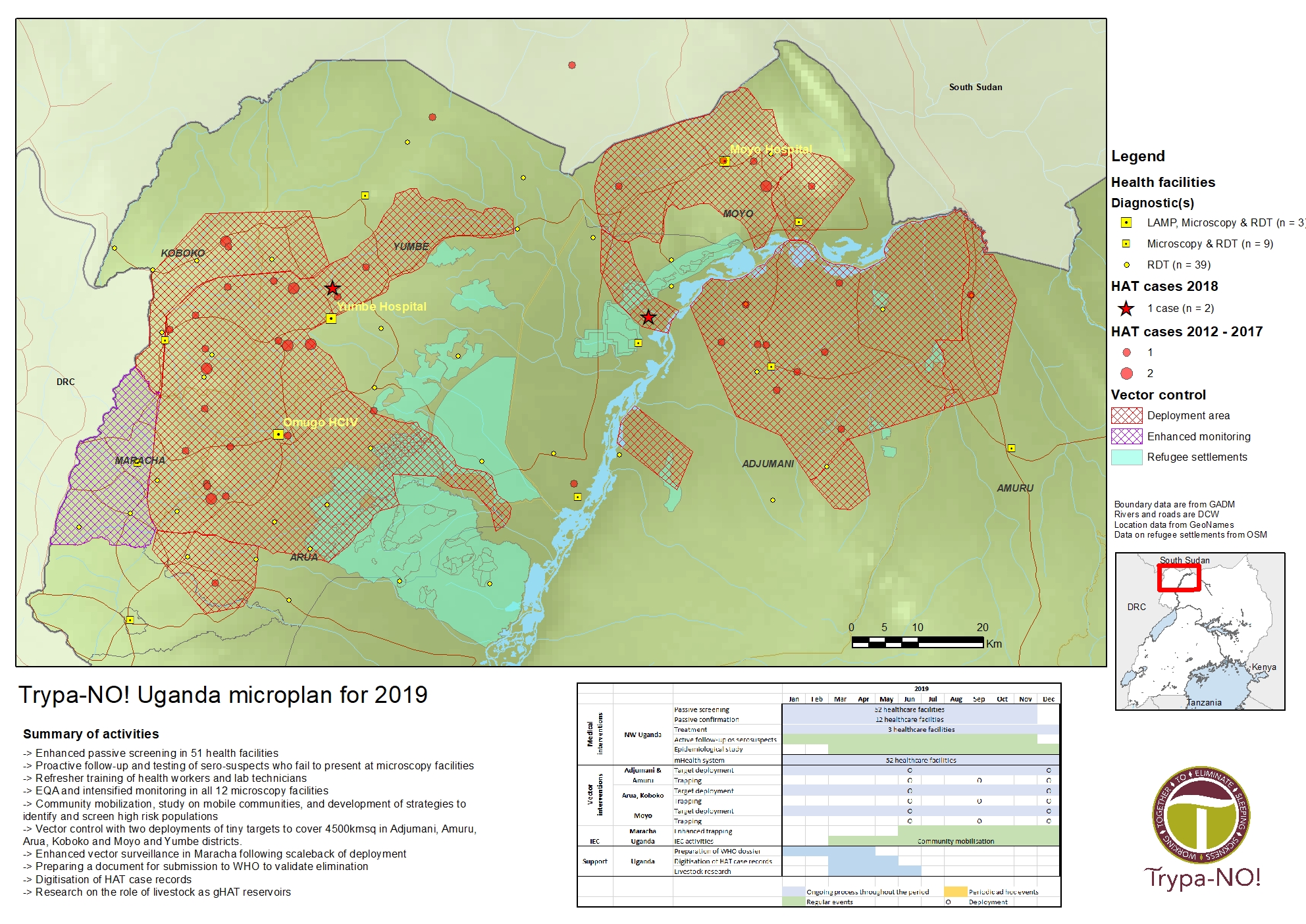

Supplement: S4 Appendix — The microplan comprises of a map showing the region where activities are ongoing and a Gantt chart describing planned activities. (JPG) [file pntd.0008738.s006.jpg]
